# Periodontitis and the Risk of Heart Failure:a Meta-analysis and Mendelian Randomisation Study

**DOI:** 10.3290/j.ohpd.c_1793

**Published:** 2025-03-06

**Authors:** Yujia Chen, Rui Rao, Xiaozheng Wu, Zhong Qin, Yunzhi Chen, Qian Li, Wen Li

**Affiliations:** a Yujia Chen Research Assistant and Doctorate Student, Second Affiliated Hospital, Guizhou University of Traditional Chinese Medicine, Guiyang, China. Drafted, revised and approved the manuscript.; b Rui Rao Researcher, Second Affiliated Hospital, Guizhou University of Traditional Chinese Medicine, Guiyang, China. Drafted, revised and approved the manuscript.; c Xiaozheng Wu Researcher, School of Preclinical Medicine, Guizhou University of Traditional Chinese Medicine, Guiyang, China. Performed statistical analyses and interpretation, revised and approved the manuscript.; d Zhong Qin Researcher, School of Preclinical Medicine, Guizhou University of Traditional Chinese Medicine, Guiyang, China. Performed statistical analyses and interpretation, revised and approved the manuscript.; e Yunzhi Chen Professor, School of Preclinical Medicine, Guizhou University of Traditional Chinese Medicine, Guiyang, China. Contributed to data collection, revised and approved the manuscript.; f Qian Li Researcher, School of Preclinical Medicine, Guizhou University of Traditional Chinese Medicine, Guiyang, China. Contributed to data collection, revised and approved the manuscript.; g Wen Li Associate Professor, School of Preclinical Medicine, Guizhou University of Traditional Chinese Medicine, Guiyang, China. Contributed to the study design, revised and approved the manuscript.

**Keywords:** causal relationship, heart failure, Mendelian randomisation study, meta-analysis, periodontitis, systematic review

## Abstract

**Purpose:**

Periodontitis and heart failure (HF) impact millions of individuals globally with heavy social and economic burden. Prior research has indicated a connection between them. However, the conclusions have been somewhat inconsistent. Our objective is to confirm, through meta-analysis and Mendelian randomisation studies, whether patients with periodontitis have an increased risk of HF. Therefore, we conducted a comprehensive analysis to explore the causal association between periodontitis and the risk of HF.

**Materials and Methods:**

In this meta-analysis, we searched online to identify studies involving periodontitis on the risk of HF. The main endpoint assessed in this study was the risk of HF. We used R language to calculate the pooled results and create plots. A random-effects model was employed in the analyses. In the Mendelian randomisation (MR) analyses, we obtained data from public databases. MR analyses were conducted using genome-wide association data for acute and chronic periodontitis. Independent genetic variants associated significantly with each exposure (P < 5*10^-6^) were considered as instruments. The primary analysis employed the inverse variance weighted (IVW) method, which was subsequently supplemented by a series of sensitivity analyses to ensure the robustness and reliability of the findings.

**Results:**

Our meta-analysis included three publications, with a total of 21,997 participants. The pooled result demonstrated that periodontitis increased the risk of HF (OR = 1.62, 95% CI 1.29–2.03). Periodontitis increased the risk of heart failure with reduced ejection fraction (HFrEF) with a low level of heterogeneity (OR = 1.99, 95% CI 1.22–3.23) and heart failure with preserved ejection fraction (HFpEF) with little heterogeneity (OR = 1.36, 95% CI 1.00–1.86). In the MR study, acute or chronic periodontitis did not increase the risk of HF. Sensitivity analyses revealed that the causal association estimations were robust.

**Conclusion:**

In summary, the meta-analysis results indicate that individuals with periodontitis are at a higher risk of HF. The findings from the MR study fail to establish a causal link between the two variables under investigation. To validate this assertion and elucidate the fundamental mechanism, additional research is imperative.

Clinical Significance: Based on the current evidence, it cannot be concluded that there is a causal relationship between acute or chronic periodontitis and HF.

Periodontitis, a prevalent oral health condition, is characterised by a bacterial infection that leads to inflammation and subsequent damage to the gingival tissues. If left untreated, this destructive process can extend to the underlying alveolar bone, resulting in potential loss of tooth support and eventual tooth loss.^28^ Without treatment, periodontitis can lead to tooth loss or loosening. Severe periodontitis affects nearly eight million people worldwide.^30^ The global burden of severe periodontitis has been increasing over the past several decades.^5,6^

Heart failure (HF) is not a disease but a syndrome, which is a combination of signs and symptoms caused by the failure of the heart to pump blood to support the circulatory system.^15,16^ The older population could be more affected by HF.^29^ Heart failure is a major cause of morbidity and mortality, and causes a lot of healthcare-related costs, posing a huge burden on patients and society.^25^ HF can be graded by ejection fraction with preserved ejection fraction (HFpEF), mildly reduced ejection fraction (HFmrEF) and reduced ejection fraction (HFrEF).^22^

The literature reveals a significant association between periodontal disease and an elevated risk of heart failure, as demonstrated in previous investigations.^19,30^ Periodontitis is increasingly recognised as an independent risk factor for cardiovascular disease.^3^

However, their conclusions conflicted slightly. In this case, a systematic review and Mendelian randomisation study may help draw a more comprehensive conclusion. We seek to gain a clearer understanding of the causal relationship between various types of periodontal disease and the presence of HF. Hence, the primary objective of this analysis is to determine the causal impact of acute and chronic periodontitis on HF.

## MATERIALS AND METHODS

### Methods for Meta-analysis

#### Protocol

The protocol of this article was registered in the international platform of registered systematic review and meta-analysis protocols (INPLASY, https://inplasy.com/), and the present systematic review was written following PRISMA^18^ (Preferred Reporting Items for Systematic Reviews and Meta-Analyses) guidelines. The registration number is INPLASY2022110084 and the DOI number is 10.37766/inplasy2022.11.0084.

#### Search strategy

A systematic literature review was conducted to identify original clinical studies published before August 2024 in the Cochrane Central Register of Controlled Trials (CENTRAL) (onlinelibrary.wiley.com/cochranelibrary/), PubMed (www.ncbi.nlm.nih.gov/pubmed), and Embase (www.embase.com) using the search term: (periodontitis [Title/Abstract] OR gingivitis [Title/Abstract] OR periodont* [Title/Abstract]) AND (heart failure [Title/Abstract]). The search methodology adhered to the guidelines outlined in the *Cochrane Handbook* and was independently carried out by two investigators. Furthermore, the references of all included articles and relevant reviews were also screened to ensure comprehensive coverage of the literature.

#### Study selection

We filtered the literature according to the following criteria: 1) observational studies (both case-control and cohort studies); 2) patient age >18 years; 3) examined the causal association of periodontitis with HF; 4) the odds ratios (ORs) or hazard ratios (HRs) of HF were reported in both periodontitis and non- periodontitis population.

The excluded articles were eliminated as the following criteria: 1) duplicate articles; 2) reviews; 3) letters to the editor; 4) case reports; 5) cell or animal research. Independently, two authors screened the titles and abstracts. If reviewers had different opinions, another reviewer was tried to be consulted to reach a consensus.

#### Data collection

Data were collected from each included article, including the author, publication year, country, study design, case number, age, gender, exclusion and inclusion criteria, outcomes, and statistical methods, respectively. The quality of individual publications was evaluated according to the Newcastle–Ottawa Scale.^14^

#### Outcomes

The primary outcome was the risk of HF in general. The secondary outcomes were the risk of HFrEF and HFpEF.

#### Statistical analysis

R software open-source edition (Version 4.2.3, Vienna, Austria) was used for calculating the pooled results of the included studies and plotting. R version 4.2.3 and meta-package were used. *I* ^2^ and Chi^2^ tests were performed to assess heterogeneity. Random-effects model calculation was finally used for the analysis. Odds ratios (ORs) were selected for dichotomous variables to be the effect sizes.

### Methods for Mendelian Randomisation Study

#### Study design

In an effort to evaluate the causative associations between periodontitis and the risk of HF, we implemented an MR analysis. This analysis was performed by leveraging publicly accessible summary-level data derived from genome-wide association studies (GWASs). The following critical assumptions were made in this study: Instrumental variables were strongly associated with exposure and independent of confounders of exposure and outcome. Instrumental variables affected the outcome only through exposure.^9^ All data used in this study were obtained from publicly accessible sources, and therefore, there were no ethical conflicts. The findings of this study were reported following the Strengthening the Reporting of Observational Studies in Epidemiology Using Mendelian Randomisation (STROBE-MR) guidelines.^26^

#### GWAS summary data for periodontitis and genetic instruments selection

Genetic association estimates of single nucleotide polymorphisms (SNPs) with acute and chronic periodontitis were obtained from the Integrative Epidemiologic Unit (IEU) open GWAS project (https://gwas.mrcieu.ac.uk/).^7^ Two data sets of acute and chronic periodontitis from FinnGen were included in our study (Table 3). FinnGen represents a substantial public–private collaborative endeavour with the objective of amassing and scrutinising genomic and health-related data derived from a cohort of 500,000 participants enlisted from Finnish biobanks.^10^ In accordance with the TwoSampleMR package guidelines,^2,21^ we established the following criteria for selecting suitable genetic instrumental variables: SNPs associated with each exposure (acute and chronic periodontitis) must meet a genome-wide significance threshold (*P *<5*10^–8^). If no SNPs satisfy this threshold, it will be adjusted to 5*10^–6^. To prevent linkage disequilibrium, we conducted a clumping procedure with R^2^ < 0.001 and a clumping window of 10,000 kb. We excluded SNPs that displayed a significant association with heart failure (*P* < 5*10^–8^). We incorporated SNPs with F-statistics > 10, indicating that the genetic variants possessed relatively strong estimated effects. Essential information for the SNPs, including the effect allele, other allele, β, se, and *P* value, was systematically gathered for subsequent analysis.

**Table 3:** Characteristics of the data used in the MR study

Trait	ID on open GWAS project	Data source	Year	Population	Gender	n case	n control
**Exposures**							
Acute periodontitis	finn-b-K11_PERIODON_ACUTE	FinnGen	2021	European	Males and Females	367	195,395
Chronic periodontitis	finn-b-K11_PERIODON_CHRON	FinnGen	2021	European	Males and Females	3,046	195,395
**Outcomes**							
Heart failure	ukb-d-I50	UK Biobank	2018	European	Males and Females	1,088	360,106
Heart failure	finn-b-I9_HEARTFAIL_ALLCAUSE	FinnGen	2021	European	Males and Females	23,397	194,811


#### GWAS summary data for heart failure

Genetic determinants of HF were obtained from summary-level GWAS results in the UK Biobank and FinGen, publicly available in the Integrative Epidemiologic Unit (IEU) GWAS database (Table 3).

#### Mendelian randomisation analysis

In this study, we utilised five distinct methods to assess the causal impact of acute and chronic periodontitis on the risk of heart failure. These methods included random-effects inverse variance weighted (IVW), MR-Egger regression, weighted median, simple mode, and weighted mode approaches. Among these, the IVW method served as the primary analysis tool to evaluate the causal relationships between the exposure and outcome variables, as it is the most commonly used approach in MR studies and offers the most accurate results when all selected SNPs are considered valid instrumental variables. The remaining four methods were employed as supplementary techniques for the MR analysis.

#### Sensitivity analyses

The sensitivity analyses included tests for heterogeneity and genetic pleiotropy, leave-one-out analysis, and a funnel plot. Firstly, we calculated the Cochran’s Q statistic to estimate the heterogeneity of the IVW approach. The *P* value of Cochran’s Q test was utilised to test for heterogeneity, with a *P* value of less than 0.05 indicating heterogeneity. Secondly, we used the intercept from MR-Egger regression to estimate genetic pleiotropy, specifically examining horizontal pleiotropy. A *P* value of less than 0.05 indicated the presence of horizontal pleiotropy. Thirdly, we carried out a leave-one-out analysis by removing each SNP individually and testing the remaining SNPs to detect potential outliers. Lastly, we employed a funnel plot for a visual inspection of asymmetry, which may suggest violations of the MR assumption due to horizontal pleiotropy.

#### Statistical analysis and plot

Causal estimates were displayed as OR and 95% confidence interval (CI). P value was considered statistically significant at less than 0.05. The scatter plot, leave-one-out plot, and funnel plots were conducted using the ‘’TwoSampleMR’ package (https:// mrcieu.github.io/TwoSampleMR/) in R (version 4.2.3 Project for Statistical Computing, Vienna, Austria). Forest plots were performed using ‘ForestPloter’ package (https://github.com/adayim/forestploter).

## RESULTS

### Results of Meta-analysis

#### Study description and risk of bias

**Fig 1:**
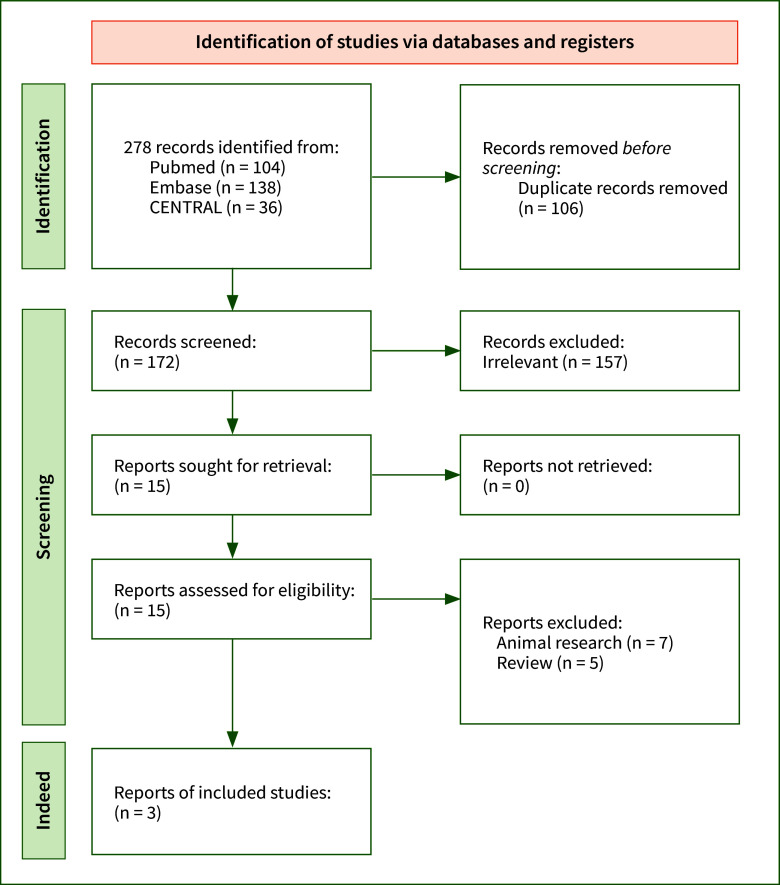
Study flow diagram.

By searching online with the methods mentioned above, a total of 172 publications were identified after duplicated articles were removed. After the title and the abstracts were checked, the full texts of 15 records were downloaded (Supplementary Table S2, S3 and S4). Three articles were ultimately used in our analysis, including a total of 21,997 patients. The study selection detail is shown in Figure 1. Three observational studies were totally included in the present analysis. The characteristics of the studies are demonstrated in Table 1. All included articles were of good quality. The Newcastle–Ottawa Scale table was used to assess the bias risks (Table 2).

**Table 1:** Characteristics of the eligible publications for meta-analysis

First author	(Year) Region	Design	Number of P/NP	Age P/NP	Gender F (M) P/NP	Inclusion and exclusion criteria	Outcomes	Statisticalmethods
Molinsky (2022)^19^	USA	Prospective cohort	4,420/ 1746	63.0 (6.0)/ 62.0 (5.0)	2,166 (2,254)/ 1,152 (594)	Inclusion: 1. All participants who completed the fourth clinic visit. Exclusion: 1. Missing information on periodontal status, HF status, important covariables, or self-reported race.	Adjusted HRs of HF, HFrEF and HFpEF	Cox proportional hazards model
Yan^16^ (2022)	USA	Prospective cohort	2,596/ 10,606	56.90 (16.07)/ 39.62 (16.40)	1,015 (1,581)/ 5,953 (4,653)	Inclusion: 1. People older than 18 years with clinical dental examination information. Exclusion: 1. Individuals who did not have complete information about periodontal assessments and heart failure.	Adjusted OR of HF	Logistic regression models
Walther (2022)^30^	Germany	Prospective cohort	1,176/ 1,453	*66.00 [59.00, 71.00]/ 59.00 [52.00, 66.00]	460 (716)/ 878 (575)	Inclusion: 1. All participants underwent a comprehensive oral examination and transthoracic echocardiography. Exclusion: 1. Missing full periodontal examination.	Adjusted ORs of HF, HFrEF and HFpEF	Logistic regression models
P: Periodontitis; NP: Non-periodontitis* Variables are presented as median and interquartile range

**Table 2:** Risk of bias of included observational studies for meta-analysis

	Selection	Comparability	Outcome
	1) Representativeness of the exposed cohort	2) Selection of the non-exposed cohort	3) Ascertainment of exposure	4) Demonstration that outcome of interest was not present at start of study	1) Comparability of cohorts on the basis of the design or analysis	1) Assessment of outcome	2) Was follow-up long enough for outcomes to occur	3) Adequacy of follow-up of cohorts
Molinsky (2022)^19^	1	1	1	1	1	1	1	1
Yan (2022)^16^	1	1	1	1	1	1	1	1
Walther (2022)^30^	1	1	1	1	1	1	1	1


#### Primary outcome

**Fig 2:**
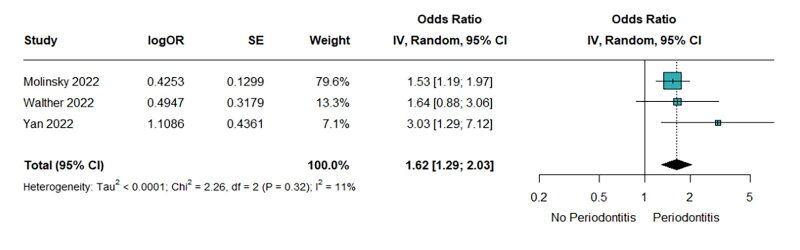
Periodontitis increased the risk of HF.

Three included studies reported the risk of HF in general as their main outcome. The pooled result showed that periodontitis increased the risk of HF among the observing population. Slight heterogeneity was observed, and the result was significant (OR = 1.62, 95% CI 1.29–2.03; Chi^2^ = 2.26, *P* = 0.32; I^2^ = 11%; Fig 2).

#### Secondary outcomes

**Fig 3:**
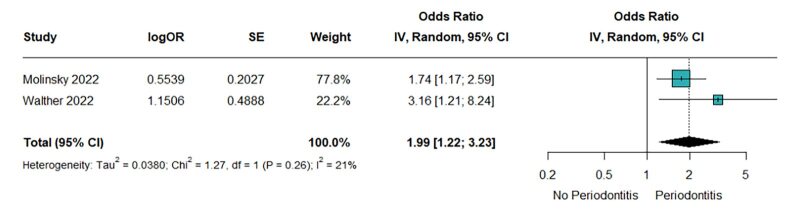
Periodontitis increased the risk of HFrEF.

Two individual studies reported the association of periodontitis with the risk of HFrEF. The pooled analysis indicated that periodontitis increased the risk of HFrEF with a low level of heterogeneity. Statistical significance was observed (OR = 1.99, 95% CI 1.22–3.23; Chi^2^ = 1.27, *P* = 0.26; I^2^ = 21%; Fig 3).

**Fig 4:**
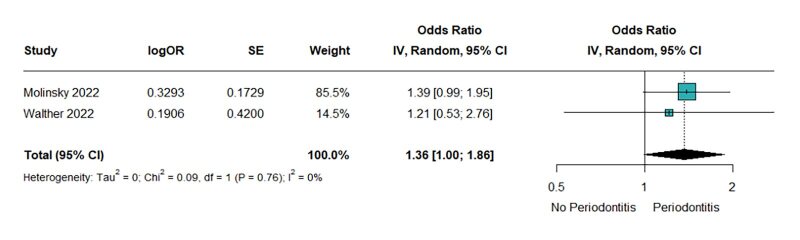
Periodontitis increased the risk of HFpEF without statistical significance.

Two publications reported the association of periodontitis with the risk of HFpEF. The forest plot demonstrated that periodontitis increased the risk of HFpEF with little heterogeneity. Unfortunately, the lower CI of the odds ratio just reached 1.0, indicating that there is no statistical significance (OR = 1.36, 95% CI 1.00–1.86; Chi^2^ = 0.09, *P* = 0.76; I^2^ = 0%; Fig 4).

### Results of Mendelian Randomisation Study

#### Characteristics of the genetic instruments

Upon completing the process of instrument selection, a total of ten SNPs were identified for the genetic prediction of acute periodontitis, while 16 index SNPs were utilised for the genetic prediction of chronic periodontitis. The F-statistics for these respective genetic instruments exceeded 10, indicating that no weak instruments were implemented in this study.

#### Mendelian randomisation analysis

**Fig 5:**
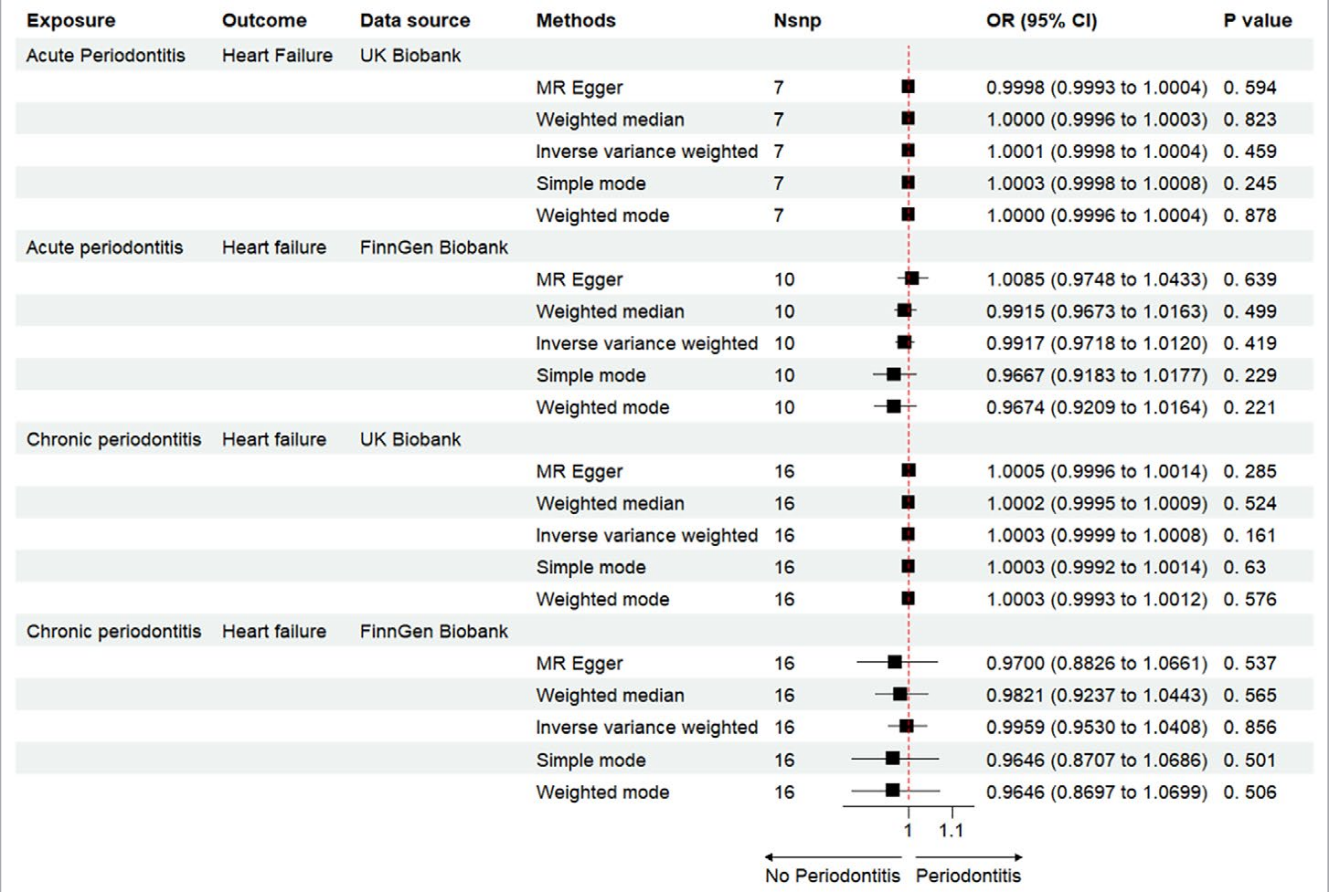
Causal effects of periodontitis on the risk of heart failure.

**Fig 6:**
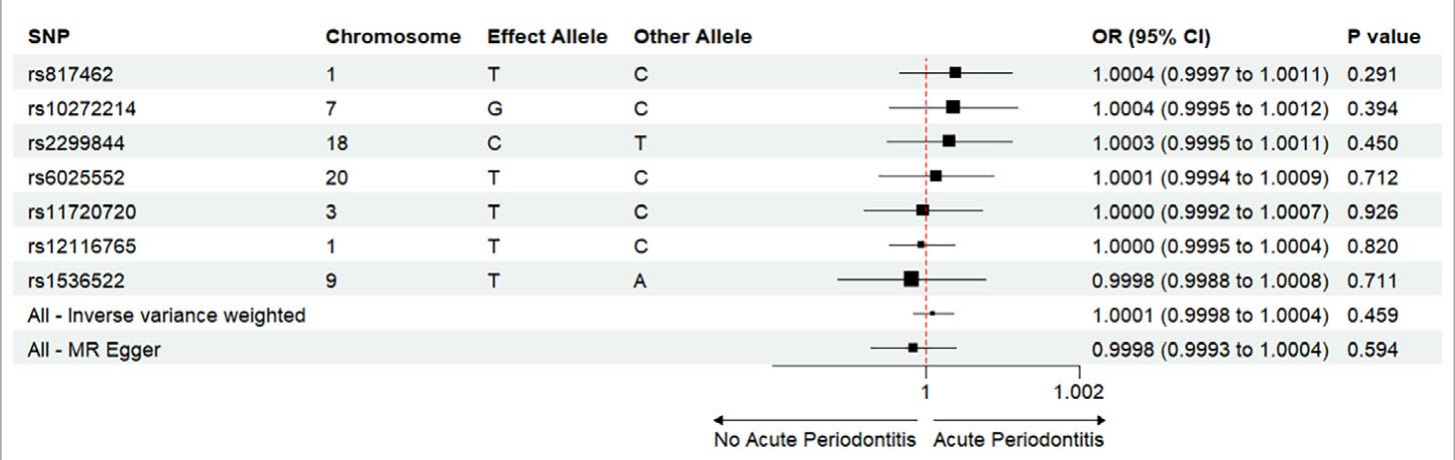
Causal effects of acute periodontitis on the risk of heart failure per allele from the data source of UK biobank.

**Fig 7:**
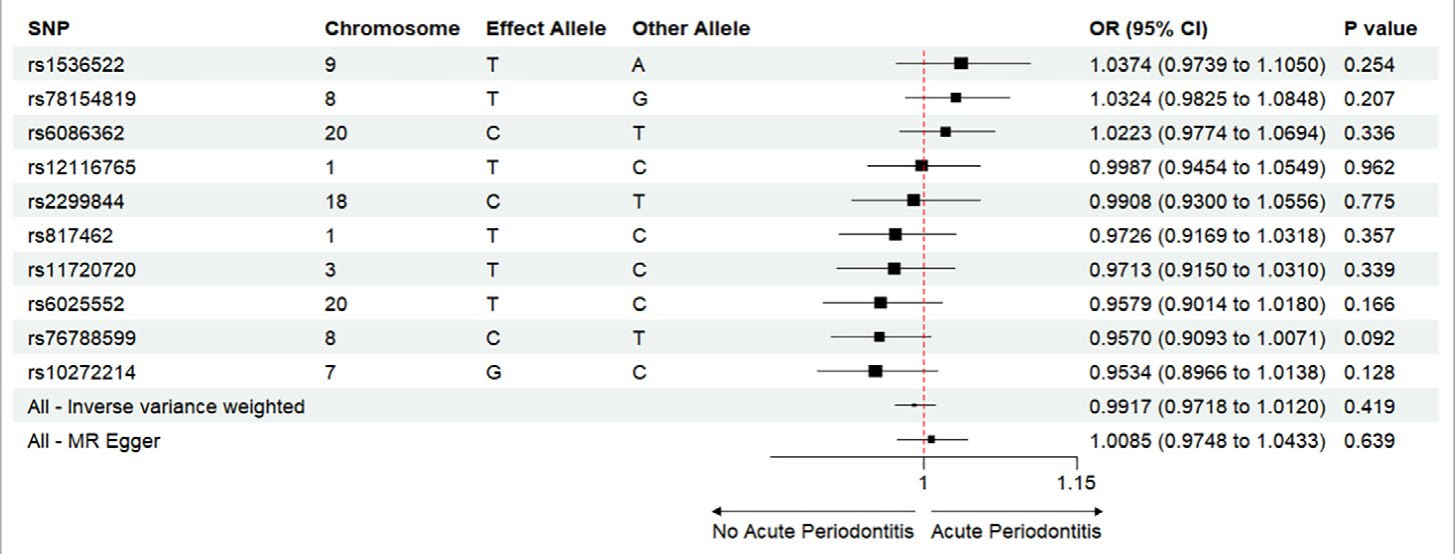
Causal effects of acute periodontitis on the risk of heart failure per allele from the data source of FinnGen.

**Fig 8:**
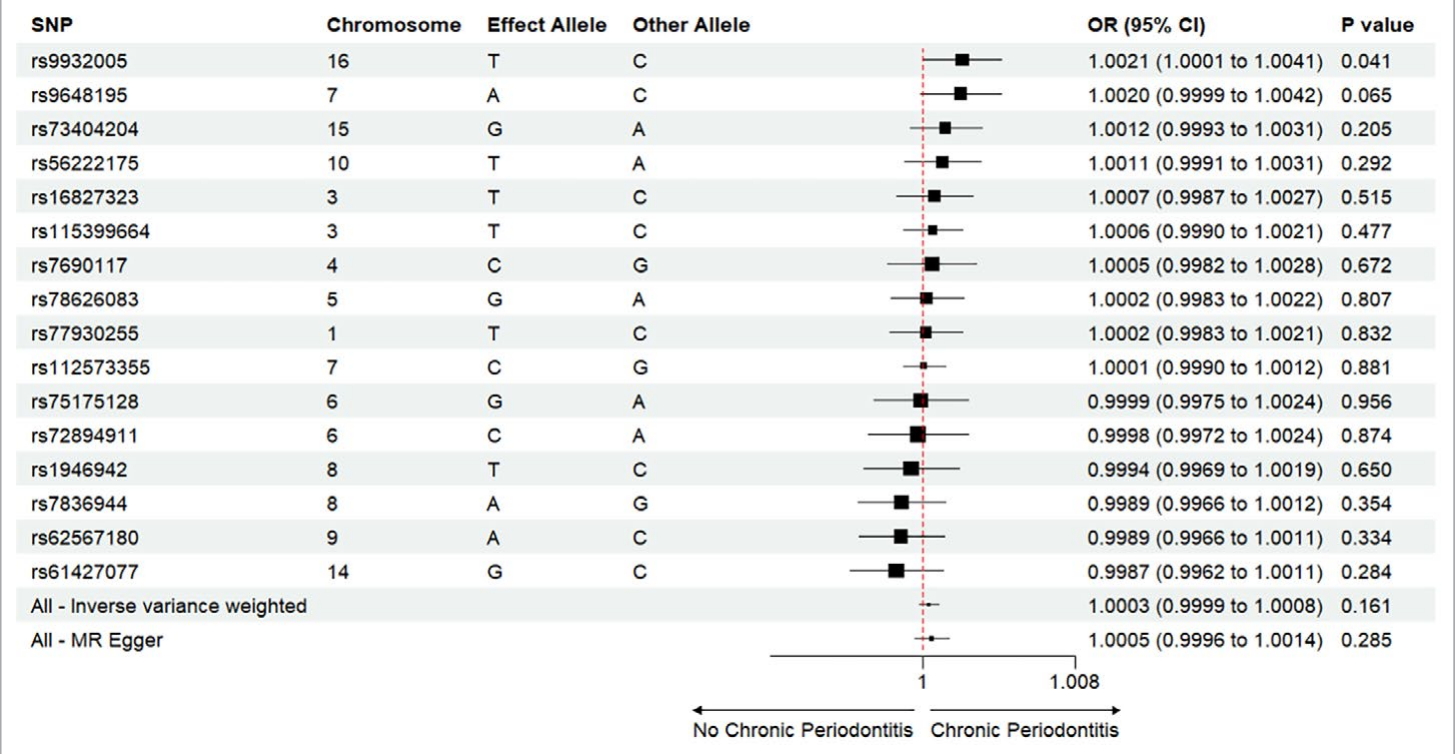
Causal effects of chronic periodontitis on the risk of heart failure per allele from the data source of UK biobank.

**Fig 9:**
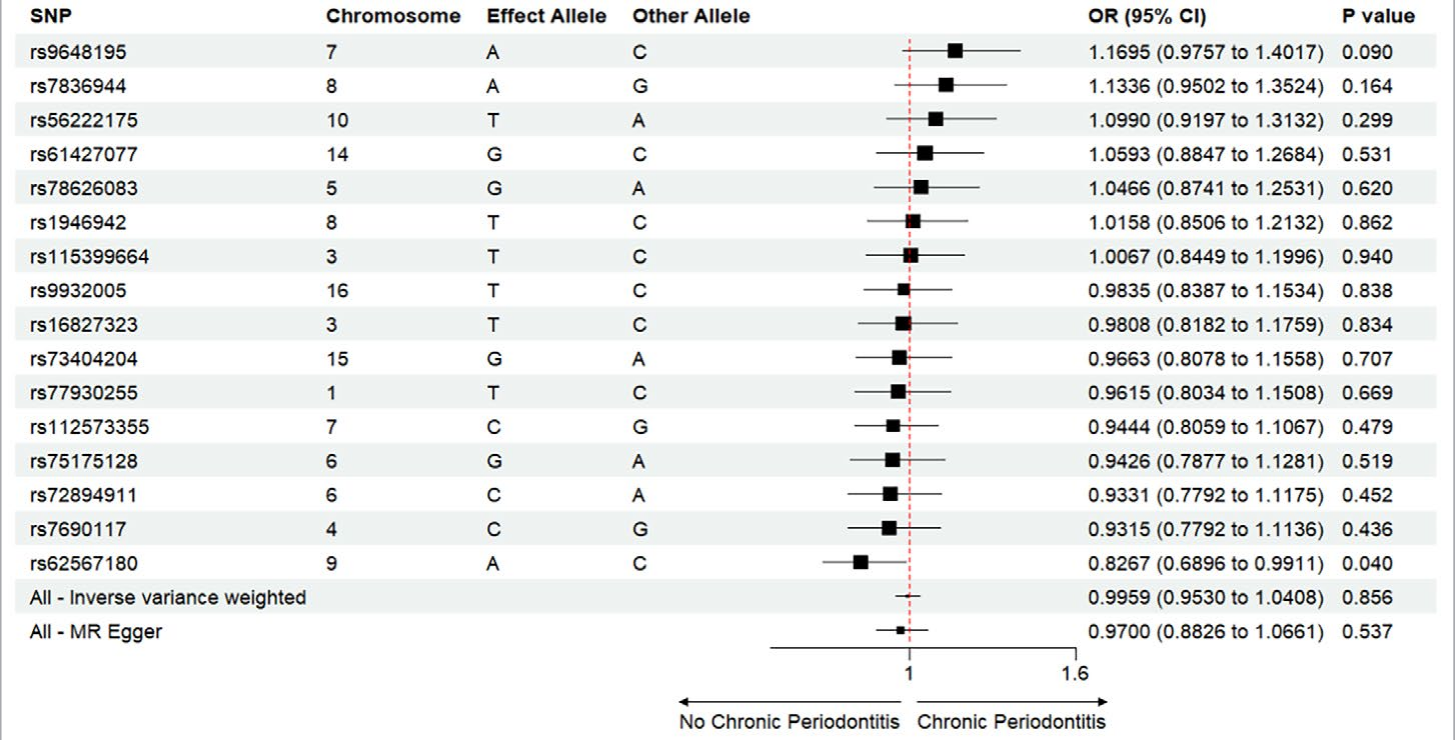
Causal effects of chronic periodontitis on the risk of heart failure per allele from the data source of FinnGen.

The Mendelian randomisation analysis presents estimates from various methods for the causal effects of acute and chronic periodontitis on the risk of heart failure, as shown in Figure 5. The results indicate that there is no significant risk change associated with heart failure for either acute or chronic periodontitis. Under the IVW method, acute periodontitis does not increase the risk of heart failure. This conclusion is consistent across both the UK Biobank and FinGen outcome data sets (OR = 1.0001, 95% CI 0.9998–1.0004; *P* = 0.459; UK Biobank) (OR = 0.9917, 95% CI 0.9718–1.0120; *P *= 0.419; FinGen). Similarly, there is no statistically significant relationship between chronic periodontitis and heart failure risk (OR = 1.0003, 95% CI 0.9999–1.0008; *P* = 0.161; UK Biobank) (OR = 0.9959, 95% CI 0.9530–1.0408; *P* = 0.856; FinGen). The scatter plots for MR analyses are demonstrated in Supplementary Figures 10 to 13. The causal effects of periodontitis on the risk of heart failure per allele are shown in Figures 6 to 9.

#### Sensitivity analyses

**Fig S19:**
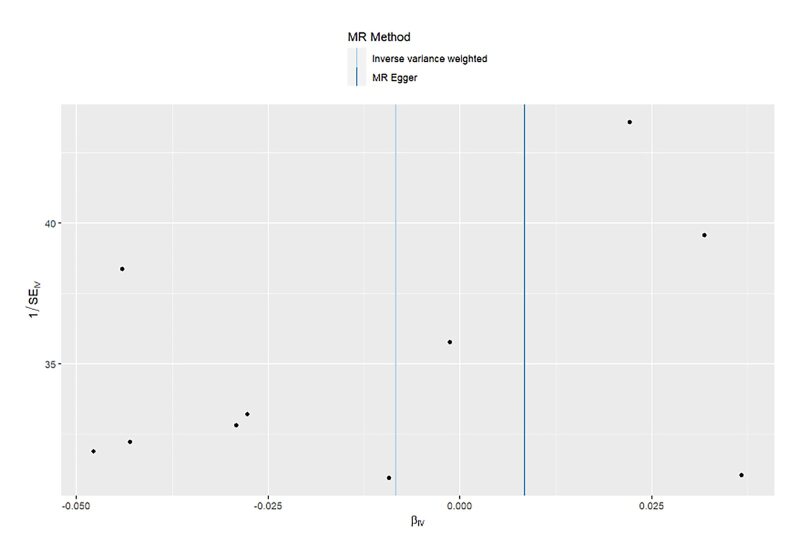
Funnel plot for MR analysis of acute periodontitis on heart failure from the database of FinGen.

**Fig S20:**
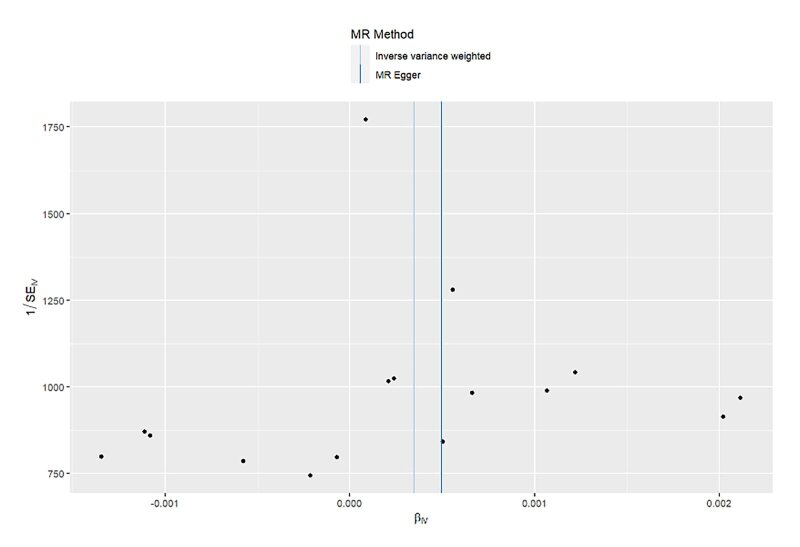
Funnel plot for MR analysis of chronic periodontitis on heart failure from the database of UK biobank.

**Fig S21:**
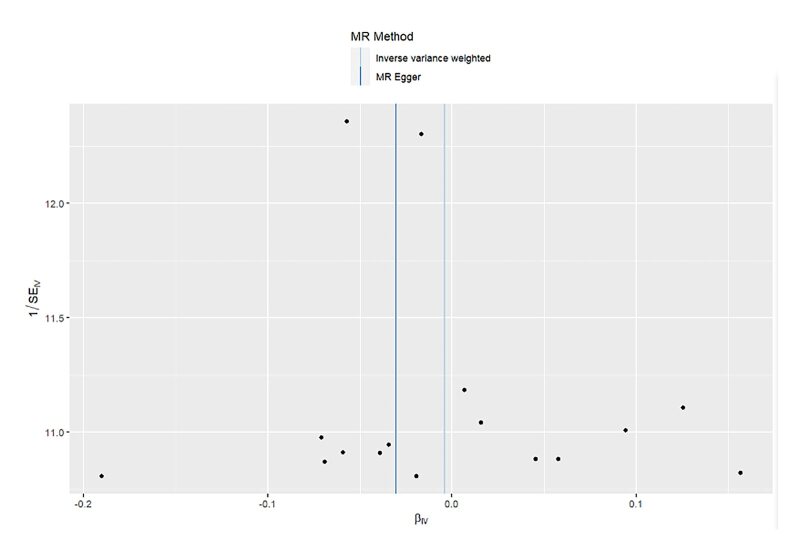
Funnel plot for MR analysis of chronic periodontitis on heart failure from the database of FinGen.

In order to evaluate the reliability of our findings, we performed a series of analyses, which included Cochran’s Q test, the MR-Egger intercept test, leave-one-out analysis, and a funnel plot. The results of the MR-Egger intercept test and Cochran’s Q test can be found in Supplementary Table S1. There was no evidence of horizontal pleiotropy between the instrumental variables and outcomes, as all *P* values of the MR-Egger intercept tests were greater than 0.05. Furthermore, no heterogeneity was detected in the Cochran’s Q test analysis, with all *P* values of the Cochran’s Q tests exceeding 0.05. The leave-one-out analysis demonstrated that the causal estimates of periodontitis and the risk of heart failure were not influenced by any single SNP, as shown in Supplementary Figures 14 to 17. Finally, the funnel plots for the MR analysis revealed an equal distribution of data points around the funnel, suggesting the absence of significant asymmetry (Supplementary Figs 18 to 21).

## DISCUSSION

In this comprehensive meta-analysis of three cohort studies, a total of 21,997 participants were included. The findings revealed a significant association between periodontitis and HF (OR = 1.62, 95% CI 1.29–2.03; Chi^2^ = 2.26, *P* = 0.32; I^2^ = 11%). As well as its subtype, heart failure with reduced ejection fraction (HFrEF) (OR = 1.99, 95% CI 1.22–3.23; Chi^2^ = 1.27, *P* = 0.26; I^2^ = 21%). The results for heart failure with preserved ejection fraction (HFpEF) were not statistically significant (OR = 1.36, 95% CI 1.00–1.86; Chi^2^ = 0.09, *P* = 0.76; I^2^ = 0%).

However, in our Mendelian randomisation (MR) study, we did not identify any significant risks associated with acute or chronic periodontitis in relation to heart failure.

Based on the available literature, there are possible mechanisms at play in the relationship between periodontal disease and heart failure. These mechanisms may serve as potential links between periodontal disorders and cardiovascular diseases. Periodontitis is an inflammation of the periodontium, which is often caused by poor oral hygiene habits.4 Improved oral hygiene care was associated with decreased systemic inflammation and following risk of heart disease.^15^ Periodontal problems were associated with increased levels of NT-pro-BNP and C-reactive protein (CRP).^19^ In the study, it was observed that the mean ratios of neutrophil to lymphocyte (NLR), platelet to leukocyte (PLR), and lymphocyte to monocyte (LMR) were significantly elevated in patients with periodontitis in comparison to the control group.^1^ IL-6, TNF-α, CRP, as well as other inflammatory biomarkers, are known to be increased in HF patients and are predictors of poor clinical outcomes.^27^ Meanwhile, treatment of periodontitis reduces serum CRP levels.^12^ The evidence indicated associations between periodontitis and inflammation. Inflammation has long been proven to have an inseparable relationship with various heart diseases. The area under the curve of the systemic immune-inflammation index could even be used in predicting the severity of coronary heart disease.^11^ TNF-a promotes cardiac apoptosis and fibrosis, which leads to a direct negative systolic and diastolic effect.^31^ Increased levels of IL-6 have been observed in patients with acute myocarditis and are associated with poor prognosis.^24^ The translocation of bacterial lipopolysaccharide (LPS) into the bloodstream leads to the development of endotoxemia, a condition that is known to be associated with an elevated risk of cardiometabolic disorders. While the primary source of endotoxemia is commonly believed to be the intestinal microbiota, it is worth considering that the dysbiotic periodontal microbiota may also contribute to the occurrence of endotoxemia in patients diagnosed with periodontitis.^8^ Chronic endotoxemia has been implicated in the pathogenesis of several inflammation-driven diseases, particularly cardiovascular and metabolic disorders. The main source of endotoxemia is thought to be the gut microbiota. However, it is important to note that dysbiosis of oral microbiota, typically characterised by an abundance of Gram-negative bacteria in periodontitis, may also contribute to endotoxemia.^23^ Extracellular vesicles, specifically exosomes, represent a subset of the smallest cell-secreted extracellular-signalling vesicles that possess the ability to engage in long-distance communication with various tissues and cell types. Recently, there has been a significant increase in the utilisation of exosomes in the field of dentistry. Several studies have yielded promising results, demonstrating the potential effects of exosomes in reducing the count of periodontal pathogens and minimising the incidence of HF.^17^

Heart failure is the potential end stage of all heart diseases.^7^ Common causes of heart failure include coronary artery disease, high blood pressure, atrial fibrillation, and other unknown causes.^20^ Thus, heart failure gradually occurs and develops. However, the present Mendelian randomisation study has not identified a causal relationship between periodontitis and heart failure, as demonstrated in epidemiological research. This may be attributed to the possibility that, despite adjusting for multiple variables, we cannot completely exclude the presence of potential confounding factors. At this time, we are unable to identify what these specific confounding factors might be. This may be an area that warrants further investigation in the future.

Several limitations must be acknowledged in the present article. Firstly, only three studies were ultimately included, which may be considered insufficient. Consequently, subgroup analysis, sensitivity analysis, and publication bias evaluation could not be conducted. Secondly, the criteria for diagnosing heart failure varied slightly across different literature sources, potentially introducing bias. Thirdly, the Molinsky 2022 article^19^ carries significant weight in synthesising the pooled effect, which could also contribute to bias. The robustness of the conclusions drawn in this review should be further verified. Furthermore, in the latest 2017 classification system for periodontal diseases, the use of terms such as ‘acute periodontitis’ and ‘chronic periodontitis’ is no longer recommended. However, due to the limitations of the databases from which we obtained data, we have still utilised the classification system based on acute and chronic periodontal disease. Lastly, our findings from the MR analyses relied on data from GWASs conducted solely on individuals of European ancestry, lacking ancestral and cultural diversity. As a result, it remains uncertain whether these findings can be generalised to other ethnic groups.

## CONCLUSION

In summary, the meta-analysis results indicate that individuals with moderate/severe periodontitis are at a higher risk of heart failure. The findings derived from the MR study indicate the absence of a causal link between periodontitis and cardiac failure. We speculate that confounding factors may have led to biased results in epidemiological studies. Further research is needed to confirm this conclusion, and the underlying mechanism is still not fully understood.

### Acknowledgements

The content and charts of this article were independently completed by the listed authors. We want to acknowledge the participants and investigators of the FinnGen study.

#### Funding

Natural Science Foundation of Guizhou Province Health Commission (2022XMSB00035072), Youth Sailing Project of Guizhou University of Traditional Chinese Medicine (GZYKH-QNYFZK[2024]18) and the National Natural Science Foundation of China (No: 82260890).

#### Declarations

##### Ethical approval

Only publicly available deidentified data were utilised in this study, with no need of separate ethical approval.

##### Consent for publication

Not applicable.

##### Competing interests

The authors declare no competing interests.

##### Conflict of interest

The authors declare that they have no conflict of interest.

## SUPPLEMENTS

**Fig 10:**
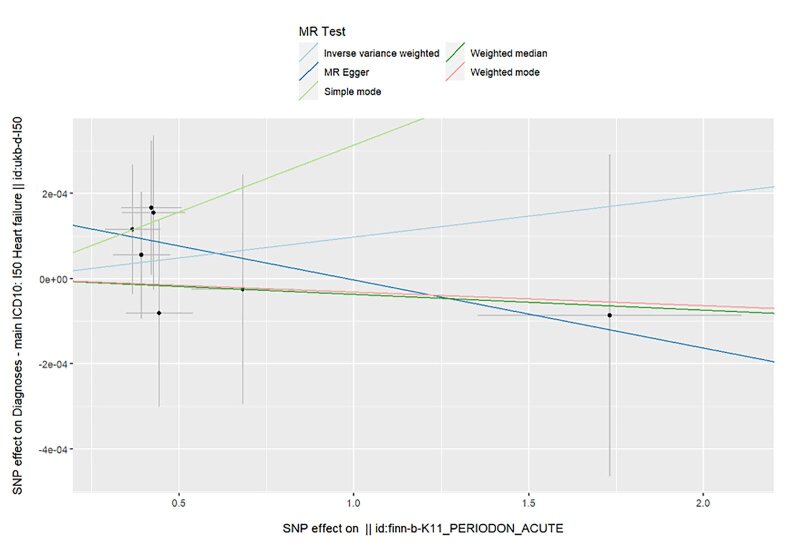
Scatter plot for MR analysis of acute periodontitis on heart failure from the database of UK biobank.

**Fig 11:**
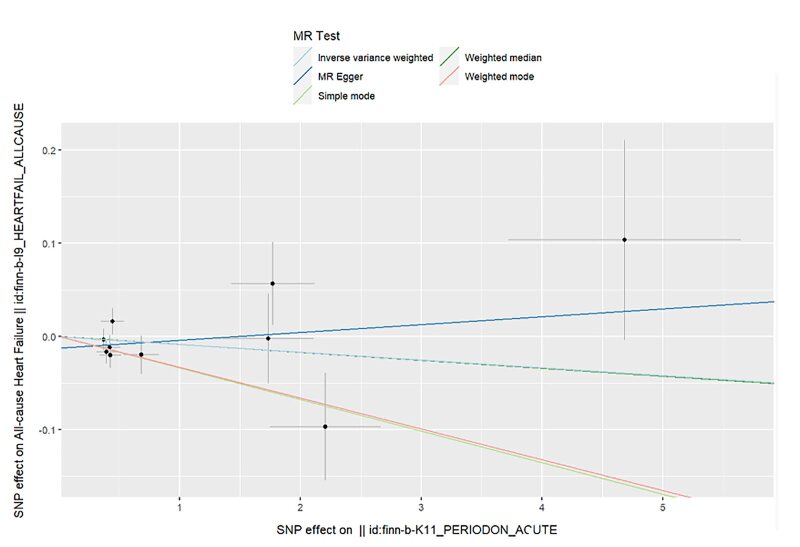
Scatter plot for MR analysis of acute periodontitis on heart failure from the database of FinGen.

**Fig 12:**
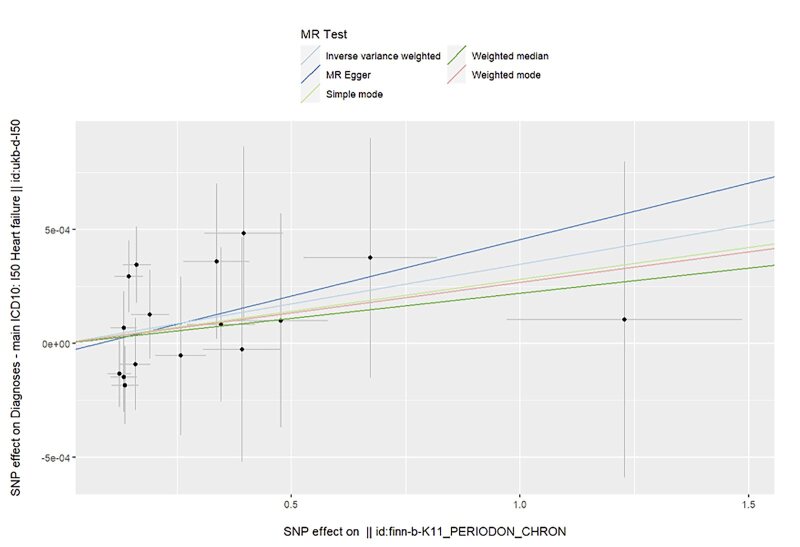
Scatter plot for MR analysis of chronic periodontitis on heart failure from the database of UK biobank.

**Fig 13:**
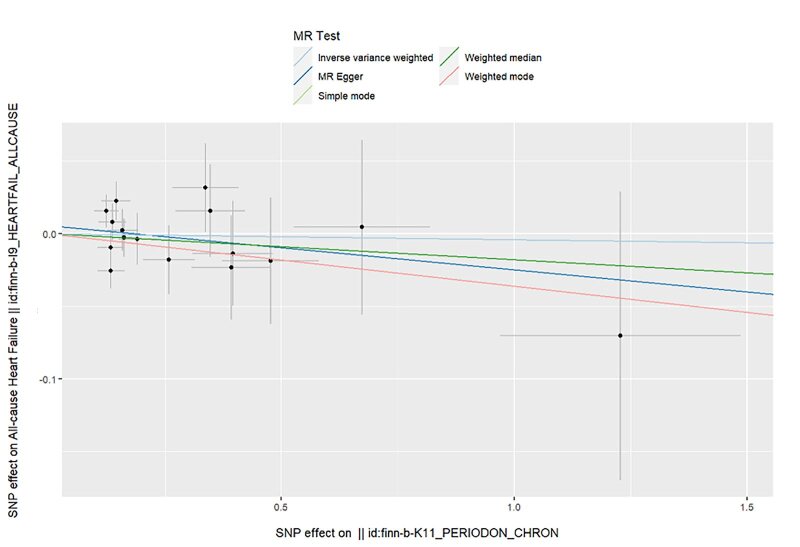
Scatter plot for MR analysis of chronic periodontitis on heart failure from the database of FinGen.

**Fig 14:**
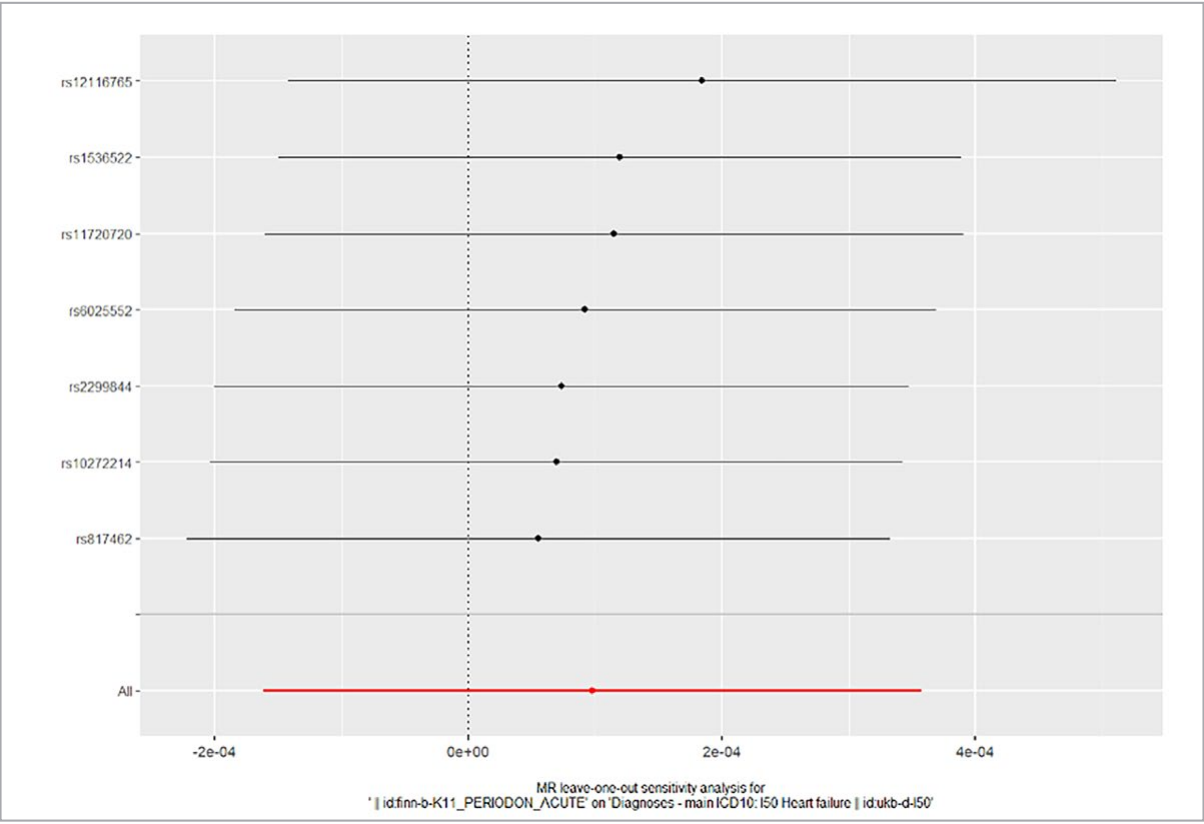
Forest plot for leave-one-out analysis of acute periodontitis on heart failure from the database of UK biobank.

**Fig 15:**
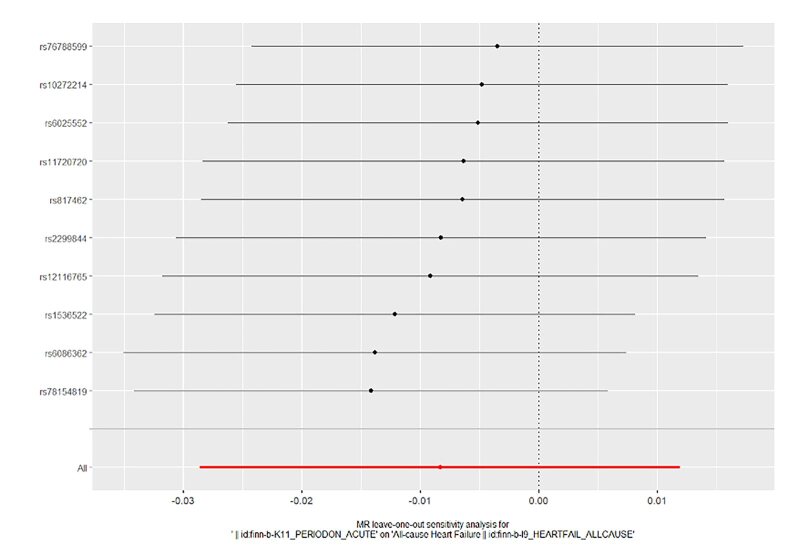
Forest plot for leave-one-out analysis of acute periodontitis on heart failure from the database of FinGen.

**Fig 16:**
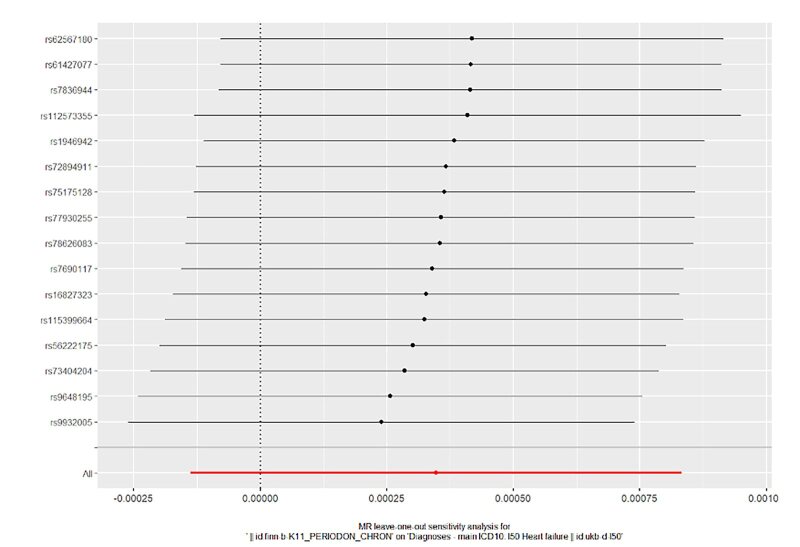
Forest plot for leave-one-out analysis of chronic periodontitis on heart failure from the database of UK biobank.

**Fig 17:**
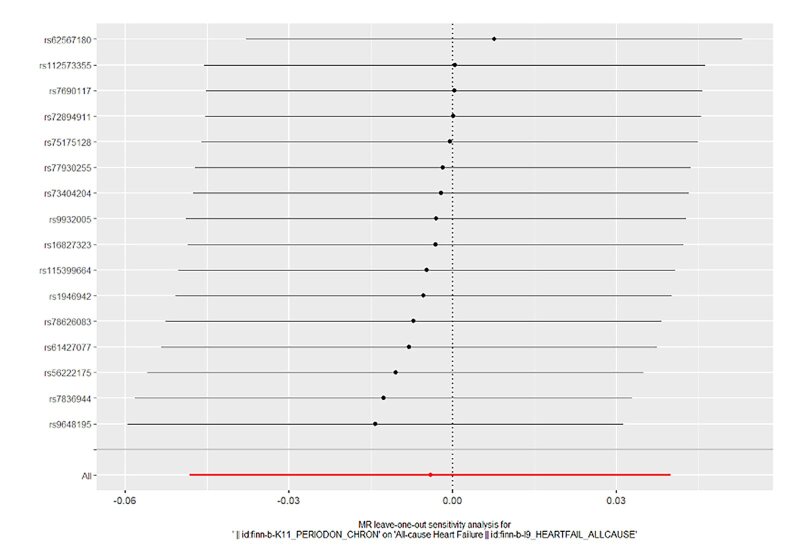
Forest plot for leave-one-out analysis of chronic periodontitis on heart failure from the database of FinGen.

**Fig 18:**
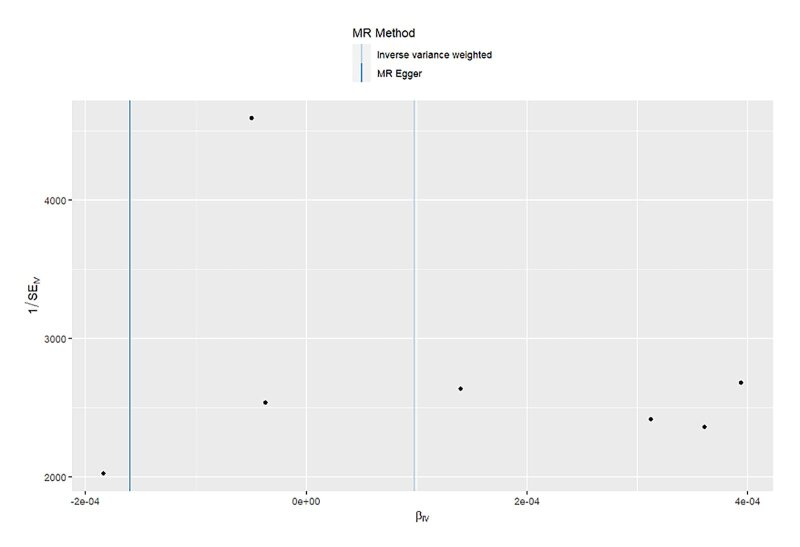
Funnel plot for MR analysis of acute periodontitis on heart failure from the database of UK biobank.

**Table S1:** Heterogeneity and pleiotropy test

Q	Q_df	*P* value	Egger Intercept	se	*P* value
Exposure	Outcome	Data source	Method	Heterogeneity test	Pleiotropy test
Acute periodontitis	HF	UK biobank	IVW	2.196	6	0.901	0.00016	0.00015	0.346
Acute periodontitis	HF	FinGen	IVW	11.88588	9	0.220	–0.01245	0.01044	0.267
Chronic periodontitis	HF	UK biobank	IVW	12.75722	15	0.621	–0.00004	0.00010	0.695
Chronic periodontitis	HF	FinGen	IVW	13.25734	15	0.582	0.00576	0.00930	0.546


**Table S2:** Excluded literature and reasons

PMID	Title	Reason
34757204	Effects of chronic *Porphylomonas gingivalis* lipopolysaccharide infusion on cardiac dysfunction in mice	Animal research
32759964	Mzb1 protects against myocardial infarction injury in mice via modulating mitochondrial function and alleviating inflammation	Animal research
32210715	Connective tissue growth factor (CTGF) regulates the fusion of osteoclast precursors by inhibiting Bcl6 in periodontitis	Animal research
27604343	A periodontal pathogen *Porphyromonas gingivalis* deteriorates Isoproterenol-Induced myocardial remodelling in mice	Animal research
27038230	Biochemical and histopathologic analysis of the effects of periodontitis on left ventricular heart tissues of rats	Animal research
30797445	Aortocavitary fistula secondary to vegetative endocarditis in a rabbit	Animal research
27859254	Influence of experimental periodontitis on cardiac oxidative stress in rats: a biochemical and histomorphometric study	Animal research
35686242	Periodontitis and cardiovascular disease: a literature review	Review
29903685	Periodontal disease, systemic inflammation and the risk of cardiovascular disease	Review
30951625	Oral consequences of obesity and metabolic syndrome in children and adolescents	Review
32489774	Periodontitis and Cardiovascular Diseases. Consensus Report	Review
7638766	Periodontal disease in adult insulin-dependent diabetics	Review


**Table S4:** Supplementary characteristics of the eligible publications for meta-analysis

First author(Year)	Region	Design	Medium follow-up time (years)	Data of the primary and secondary outcomes	Confounding factors
Molinsky (2022)^19^	USA	Prospective cohort	13	Primary outcome: incidence of HF Secondary outcomes: incidence of the HF subtypes	Baseline age, gender, race/centre, education, insurance, cigarette status, physical activity, BMI, LDL, hypertension medication, CHD, diabetes, SBP
Yan^16^ (2022)	USA	Prospective cohort	Unknown	Primary outcome: incidence of HF	Gender, age, race, body mass index, poverty income ratio, education, marital status, smoking status, drinking status, hypertension, diabetes, stroke, and asthma
Walther (2022)^30^	Germany	Prospective cohort	10.5	Primary outcome: incidence of HF Secondary outcomes: incidence of the HF subtypes	Age, sex, body mass index, smoking, diabetes, hypertension, atrial fibrillation, and coronary artery disease


**Table S3:** Forward search with the included studies

DOI or Link	Title
https://doi.org/10.1016/j.tcm.2023.03.003	Oral health as a modifiable risk factor for cardiovascular diseases
https://doi.org/10.3390/diagnostics13203184	The role of dysbiotic oral microbiota in cardiometabolic diseases: a narrative review
https://doi.org/10.1080/00015385.2023.2259192	Association between periodontal disease and heart failure: a systematic review and meta-analysis
https://doi.org/10.1016/j.cpcardiol.2024.102699	Could the periodontal therapy improve the cardiologic patient health? A narrative review
https://doi.org/10.3390/jfmk9010052	Assessment of the relationship between periodontitis and cardiac parameters in patients with early chronic heart failure: a cross-sectional study
https://doi.org/10.3389/fcvm.2023.1296405	Application of machine learning algorithms to construct and validate a prediction model for coronary heart disease risk in patients with periodontitis: a population-based study
10.1097/MD.0000000000036659	A novel nomogram for predicting risk of hypertension in US adults with periodontitis: National Health and Nutrition Examination Survey (NHANES) 2009–2014
https://doi.org/10.21270/archi.v13i1.6311	Prevalence of patients with comorbidities at the Undergraduate Clinic of Piracicaba Dental School – Brazil
10.1097/MD.0000000000034878	A systematic comparison of machine learning algorithms to develop and validate prediction model to predict heart failure risk in middle-aged and elderly patients with periodontitis (NHANES 2009–2014)
https://doi.org/10.1097/md.0000000000036659	A novel nomogram for predicting risk of hypertension in US adults with periodontitis: National Health and Nutrition Examination Survey (NHANES) 2009–2014
https://doi.org/10.1111/jcmm.18297	The role of autophagy in odontogenesis, dental implant surgery, periapical and periodontal diseases
https://doi.org/10.1016/j.healun.2024.04.069	Alterations in the sarcopenia index are associated with inflammation, gut, and oral microbiota among heart failure, left ventricular assist device, and heart transplant patients
https://doi.org/10.1016/j.jchf.2023.04.002	JACC: Heart Failure Christopher O’Connor Award for Outstanding Scholarship
DOI : 10.6261/RJOR.2024.1.16.25	The influence of oral rehabilitation on minimising risks, enhancing prognosis and therapeutic outcome in cardiovascular patients
https://doi.org/10.1016/j.jchf.2022.08.008	Does an apple a day keep the heart failure doctor away?
	The digestive tract microbiome and cardiometabolic disease: exploring nitric oxide and lipopolysaccharide synthesis as mechanistic intermediates
https://hdl.handle.net/20.500.12880/5646	La prevalencia de la enfermedad periodontal en pacientes con enfermedad cardiovascular. Revisión sistemática
https://doi.org/10.20944/preprints202305.1601.v1	The bidirectional association between periodontitis and COVID-19: a review of current evidence
https://hdl.handle.net/11299/258631	Investigating infection-related hospitalisation as a risk factor for incident heart failure and mortality among heart failure patients
10.1016/j.tcm.2023.03.005	Editorial commentary: the complex interplay between periodontal and cardiovascular disease: the eyes to know the soul, the mouth to see the heart
https://doi.org/10.1002/cphy.c230012	Human gut microbiota in cardiovascular disease
https://doi.org/10.1016/j.jdent.2023.104804	Polymorphism of salivary proteins and risk of periodontal diseases: a systematic review and meta-analysis of clinical studies
https://doi.org/10.1111/jcpe.13865	Association between probiotic consumption and periodontitis: evidence from NHANES 2009–2014
https://doi.org/10.1038/s41598-023-41009-4	Bidirectional associations between periodontal disease and systemic diseases: a nationwide population-based study in Korea
https://doi.org/10.1002/JPER.23-0277	Association between missing teeth number and all-cause and cardiovascular mortality: NHANES 1999–2004 and 2009–2014
https://doi.org/10.1161/JAHA.122.027974	Periodontal disease associated with interstitial myocardial fibrosis: the multiethnic study of atherosclerosis
https://doi.org/10.1007/s00784-024-05690-7	Association between blood ethylene oxide levels and the prevalence of periodontitis: evidence from NHANES 2013–2014
https://doi.org/10.3390/biomedicines12061341	Exploring periodontal conditions, salivary markers, and systemic inflammation in patients with cardiovascular diseases
https://doi.org/10.1186/s12872-023-03612-1	Secondary analysis of potential associations between oral health and infection-related parameters in patients with severe heart failure — results of a German cohort
DOI: https://doi.org/10.18103/mra.v10i9.3148	Oral biofilms and their connection to systemic health
https://doi.org/10.4264/numa.82.5_287	The relationship between severity of periodontitis and atherosclerotic cardiovascular status in patients with acute myocardial infarctions: a cross-sectional study
	Maladies cardiovasculaires et maladies parodontales

